# In Vivo and Ex Vivo Gene Electrotransfer in Ophthalmological Disorders

**DOI:** 10.3390/biomedicines10081889

**Published:** 2022-08-04

**Authors:** Roberta Fusco, Giacomo Perazzolo Gallo, Elio Di Bernardo, Valeria D’Alessio, Mattia Ronchetti, Matteo Cadossi, Ruggero Cadossi

**Affiliations:** 1 Oncology Medical and Research & Development Division, IGEA SpA, 41012 Carpi, Italy; 2 Regulatory Affairs, BVI—Optikon 2000, 00138 Rome, Italy

**Keywords:** gene electrotransfer, ophthalmological disorders, in vivo, ex-vivo

## Abstract

The aim of this document is to present an overview of gene electrotransfer in ophthalmological disorders. In order to ensure an adequate variety of the assessed studies, several electronic databases were considered and studies published between January 1998 and December 2021 were analysed. Three investigators carried out data extraction and analysis, focusing on both technical (i.e., electrical protocol, type of electrode, plasmid) and medical (i.e., type of study, threated disease) aspects and highlighting the main differences in terms of results obtained. Moreover, the IGEA experience in the project “Transposon-based, targeted ex vivo gene therapy to treat age-related macular degeneration” (TargetAMD) was reported in the results section. No clinical trial was found on international literature and on ClinicalTrials.gov. Twelve preclinical studies were found including in vivo and ex-vivo applications. The studied showed that electrotransfer could be very efficient for plasmid DNA transfection. Many attempts such as modification of the electric field, buffers and electrodes have been made and the optimization of electric field setting seems to be very important. Using this technique, gene replacement can be designed in cases of retinal inheritance or corneal disease and a wide range of human eye diseases could, in the future, benefitfrom these gene therapy technologies.

## 1. Introduction

The idea of gene therapy dates back more than half a century, when it was understood that nucleic acids carry genetic information [1].

Cell electroporation or electropermeabilization generally occurs when cells either in vitro or in vivo are exposed to an external electric field. The electro gene transfer (EGT) consists in the transfer of genetic material (nucleic acids, plasmids) inside the cells through the application of electrical impulses.

The electrical pulses in EGT mode perform a dual function (1). Electroporation of target cells; (2). Electrophoretic transport of genetic material across the cell membrane.

Once inside the cell, the nucleic acid will give rise to one or more proteins that will produce the desired therapeutic or prophylactic effect.

The main areas of application of the EGT are infectious diseases; oncological diseases; genetic diseases.

The transfer efficiency of the gene material is influenced both by its characteristics (molecular weight, DNA, RNA) and by those of the tissue into which it is injected (dermis, muscle, tumor nodule).

**Figure 1:**
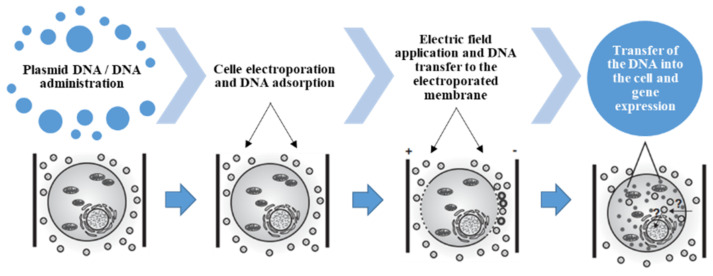
Schematic representation of gene transfer process.

Electric pulse generators can efficiently convey nucleic acids within cells allowing the user to customize the electrical protocols by varying the amplitude, duration and frequency of pulses in order to optimize the transfer of nucleic acid (Figure 1).

With the discovery of reverse transcriptase and the advent of recombinant DNA technology, the first studies began using the simian papilloma virus SV40 to correct, in vitro, symptoms of hypoxanthine–guanine phosphoribosyltransferase (HPRT) deficiency and of adenosine deaminase deficiency severe combined immunodeficiency disease (SCID). Gene therapy consists in the transfer of genes through the use of vehicles known as vectors [2].

Even though many studies are currently in progress, efficient gene delivery in human clinical trials remains a challenge. Nevertheless, different gene delivery systems have recently been designed and evaluated in order to treat both inherited and acquired disorders.

Gene delivery can take place either ex vivo or in vivo. In ex vivo approaches, the patients’ cells are collected, cultured, modified, and transplanted back to the same individual [3], while in in vivo approaches, a gene-therapy vector is directly administered to a living organism.

Different types of viral and non-viral vectors are considered, each of which has both advantages and disadvantages. Viral vectors (e.g., adenovirus, retrovirus, lentivirus and adeno-associated virus) are the most popular system and they differ from each other for cargo limits, transfection efficiency, expression, immunogenicity, and safety profile [4].

Non-viral delivery systems have some advantages, including a very low immunogenicity, a good safety profile and a potentially unlimited cargo capacity. They include naked DNA, physical agents like electroporation [5] and sonification [6], and chemical agents such as liposomes, and nanoparticles [7,8,9,10,11]. Despite non-viral methods being studied in some preclinical settings, their clinical use is challenging, as both the delivery [12,13] and transfection [14,15] are quite inefficient and the duration of expression is short in most tissues. In addition, liposomes tend to aggregate with increased risk of retinal toxicity [16] and consequently their use in retinal dystrophies is limited.

Recently, increasing attention has been paid to the applications of gene therapy in the ophthalmological field. In fact, unlike the other organs, the eye represents an immune privileged organ: (1) it is easily accessible via injections or surgically [17,18]; (2) the presence of a tight ocular barrier that permits the delivery of viral vector specifically in eye tissue preventing the diffusion in different organs and in the blood stream; (3) low doses of genetic materials are required (about 1/1000 of the amount used for systemic diseases), with a reduction of adverse reactions; (4) eyes are paired organ, thus one eye can be used as control in the same treatment; (5) assessment of the response can be done with non-invasive techniques [19,20,21].

The vectors can be administered by injection into the subretinal space or intravitreal [15]. Subretinal space is complex and requires great experience from the operator, as it involves an iatrogenic neurosensory retinal detachment near the fovea, with the risk of retinal detachment and macular hole. The intravitreal injection, on the contrary, is easier and less hazardous but less efficient for the treatment of the outer retinal disease.

Despite different strategies being available, there are some obstacles to successfully transferring this methodology to clinical applications, due to the difficulty in entering the target cells and activating the mechanism of action for a long time without adverse effects. These limitations require both the development and the choice of appropriate vectors, a crucial point that still represents a challenge for gene therapy [16]. Extensive preclinical studies have been carried out to assure feasibility, safety and efficacy. For each clinical study, permission from regulatory agency, institutional review boards and informed consent are mandatory [22]. Furthermore, ethical aspects and problem of genetic safety must be considered [23].

Electrotransfer (or electro gene transfer) is a recent application of the electroporation mechanism in gene therapy. Through the generation of electric fields, it is possible to increase cellular permeability (cell permeabilization) allowing the entry into the cell of proteins, vaccines, DNA and RNA molecules, otherwise poorly or non-permeable [24,25,26,27].

Reversible electrotransfer can be used to increase the effectiveness of basic gene therapy, with applications in various fields, such as the treatment of tumors or ocular disorders. The technique is relatively easy and safe, and has been widely used in both in vitro and in vivo applications [28,29,30]. However, its effectiveness depends on many factors, which oblige the operator to carefully plan the treatment. The choice of the electrical pulse protocol, in particular, is the aspect that has the greatest impact on the outcome of the therapy, as it has a direct correlation on the phenomenon of permeabilization [30,31,32,33,34]. In addition, a non-marginal role is played by the choice of electrodes; in fact, the shape, size and materials used not only determine the area of the target zone but also the quality of the transport, influencing the degradation of the gene material administered [30,31,32,33,34]. Finally, considering the specific contexts, the choice of the vector and the method of administration (e.g., intradermally, intramuscular, intratumoral) can have an impact in terms of outcome [29,30].

In this study, we analyzed the state of the art relating to electrotransfer applications in the ophthalmology field, in pre-clinical context, focusing in particular on the technological aspects, thus on the choice of the electrical protocol and electrodes.

## 2. Methods

This review is the result of a self-study without protocol and registration number. In order to ensure an adequate variety of the assessed studies, several electronic databases were considered: PubMed (US National Library of Medicine, http://www.ncbi.nlm.nih. gov/pubmed accessed on 7 June 2022), Scopus (Elsevier, http://www.scopus.com/ accessed on 7 June 2022), Web of Science (Thomson Reuters, http://apps.webof knowledge.com/ accessed on 7 June 2022) and Google Scholar (https://scholar.google.it/ accessed on 7 June 2022); clinicaltrials.gov (https://clinicaltrials.gov/ accessed on 7 June 2022).

Only studies published between January 1998 and December 2021 were analysed, considering this time window consistent with the recent developments concerning the fields of electrotransfer. Following keywords were considered in the research: Electrogenetransfer AND oncular; Electrogenetransfer AND Ophthalmological; gene therapy AND ocular; gene therapy AND Ophthalmological. Papers not indexed in the electronic databases were evaluated through the references of included studies. Only papers written in English and concerning the application of electrotransfer to ocular gene therapy were considered.

**Figure 2:**
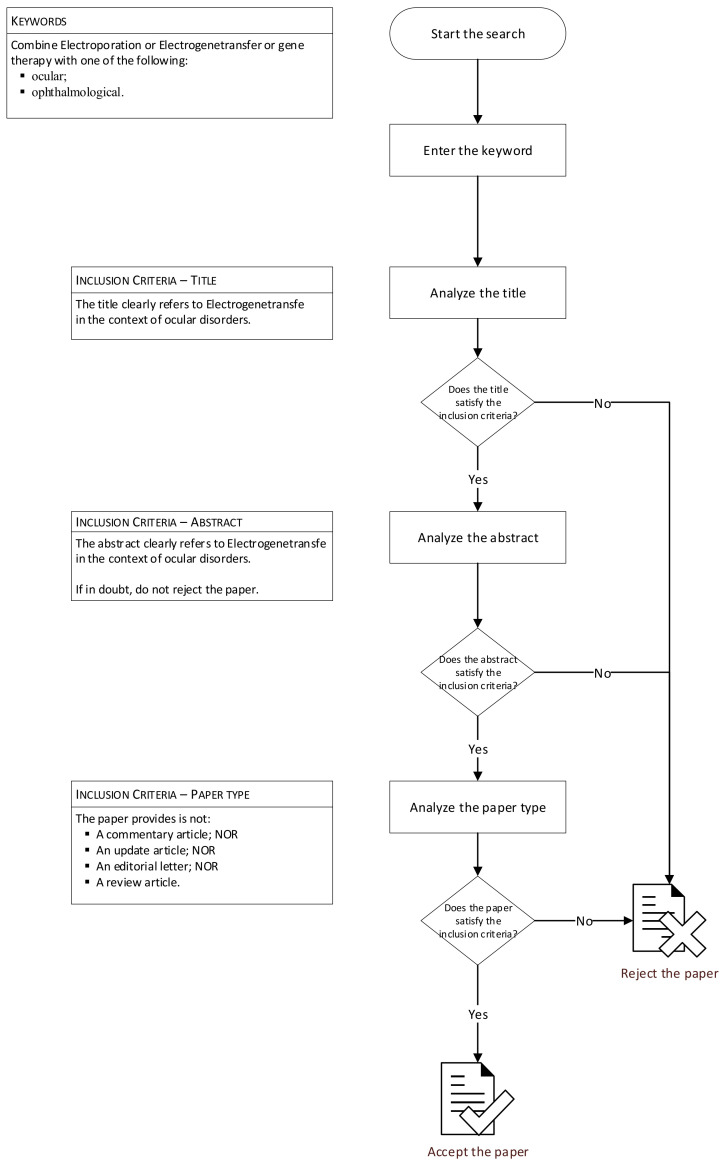
Flowchart of research methods.

Figure 2 reports the flowchart of research methods.

Three investigators carried out data extraction and analysis, focusing on both technical (i.e., electrical protocol, type of electrode, plasmid) and medical (i.e., type of study, threated disease) aspects and highlighting the main differences in terms of results obtained.

Moreover, the IGEA experience in the project “Transposon-based, targeted ex vivo gene therapy to treat age-related macular degeneration” (TargetAMD) was reported in the results section [31]. The project TargetAMD was funded by the European Commission in the context of the Seventh Framework Programme classified to the topic health. TargetAMD brings together an interdisciplinary team of outstanding scientists from 8 European countries with a broad panel of experiences and knowledge in the pathology and treatment of AMD with competences in the fields of genetic engineering, molecular and cell biology, in vivo modelling, ocular surgery, clinical studies, quality control and GMP-grade production.

## 3. Results

**Figure 3:**
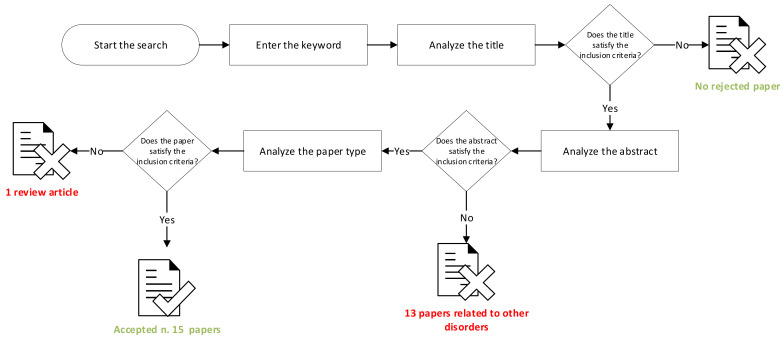
Schematic representation about included and excluded papers.

During the search, 14 documents found were excluded from the analysis due to: review article; electrogenetransfer in various applications outside of ophthalmological disorders (Figure 3). Fifteen studies were deemed suitable for the purpose of this work [32,33,34,35,36,37,38,39,40,41,42,43,44,45].

Data collected from all the papers included in the analysis are summarized in Table 1. No clinical trial was found on international literature and on clinicaltrial.gov (accessed on 7 June 2022). Exclusively preclinical studies were found including in vivo and ex vivo applications.

Oshima et al. [32] were the first to develop a method of targeted gene transfer by applying electric pulses to a selected area of corneal stroma in a rat model in vivo. A series of eight electric pulses with a pulse length of 50 ms was delivered with a standard square-wave electroporator BTX T820 (BTX, San Diego, CA, USA). The rats were divided into two groups, depending on the presence or absence of the plasmid; both groups underwent electrotransfer. Pulses with voltages ranging from 5 to 40 V/cm were delivered through a ring-shaped electrode (arrow and arrowhead). The gap distance between them was 0.5 mm. The expression of β-galactosidase was clearly detected in the cytoplasm of the corneal endothelial cells as early as day 1 and lasted until day 21. The most evident expression of β-galactosidase was achieved on day 3 in subjects treated with 20 V electric pulses. No cell damage was noticed in the cornea and no inflammation was detected in any other intraocular tissue. Thus, low-voltage electric pulses successfully transferred the gene of interest to highly selective areas of the corneal endothelium without inducing any pathological changes. This was also confirmed in their successive similar studies in rats [33,34].

Sakamoto et al. [35] investigated the therapeutic efficacy of electrotranfer in clearing the intracameral fibrin formation after bleeding. To this aim, they induced fibrin formation by means of YAG laser-generated bleeding, then they administrated tissue plasminogen activator gene to a selected area of corneal endothelium. Gene expression was enhanced applying eight square electric pulses (length of 50 ms and pause of 80 ms) through a circular stainless-steel electrode with a diameter of 0.5 mm. They found out that a biologically active tissue plasminogen activator was clearly present for 4 days after treatment, achieving a significant decrease in fibrin formation and a significantly lower corneal opacity in treated eyes than in control eyes.

In a study of 2002, Oshima et al. [36] investigate a slightly different electric protocol in order to develop a method for electrotransfer application to the corneal stroma. Using a bipolar linear stainless-steel electrode (with a 10 mm gap) placed on the surface of the cornea, they assessed the expression of the green fluorescent protein (GFP) injected into the tissue. A series of eight electric pulses with a voltage ranged from 10 to 30 V, a pulse length of 50 ms and an interval length of 75 ms were delivered. Transgene expression was detected in stromal keratocytes within the targeted area as early as day 1 after electric delivery and for the following 15 days (peaking on day 4). Gene transfer was most effective using eight electric pulses of 20 V for 50 ms without apparent cell damage in the gene transferred cells. No apparent inflammation was found in the anterior chamber or trabecular cells when electric pulses less than 30 V were used.

Electroporation of subconjunctivally and intracorneally injected plasmids (encoding luciferase or GFP) yielded equivalent high gene expression on the entire surface of the cornea in both epithelial and stromal layers in mouse model. This relatively dispersed expression, obtained by Blair-Parks et al. [37], differed from the previous studies and was explained with the use of an electrode with different design and placement. Indeed, two gold-plated rod-like electrodes, placed 3 mm apart and on each side of the protosed eye, were used to deliver eight pulses of 10 ms at 0, 100, 200, and 400 V/cm. At the field strength of 200 V/cm, gene expression was detected over the entire surface of the cornea in both epithelial and stromal layers and no trauma, corneal edema or inflammation was observed. However, at higher field strengths, corneal damage was detected.

A significant inhibition of corneal neovascularization was reported by Yu et al. [38] in an alkali burn rat model. Using the same pulse protocol and electrode as Blair-Parks et al. [37] they electroporated a subconjunctivally injected plasmid encoding the human kringle 5 of plasminogen (K5). Reverse transcription polymerase chain reaction and immunohistochemistry confirmed the expression of K5 in the conjunctiva and cornea.

In order to reduce the consequences of glaucoma-filtering surgery, especially changes of intraocular pressure (IOP) and bleb formation, Mamiya et al. [39] designed a specific study protocol to improve the effects of transfection of matrix metalloproteinase-3 (MMP-3) cDNA into rabbit conjunctiva. By delivering two series (with polarity inversion) of 5 pulses at 5 V/cm through two cup-shaped electric probes placed in both lateral sides of the bleb and before performing the glaucoma surgery, they obtained a significantly longer survival of the filtering bleb and decreased levels of IOP as compared to controls.

Chalberg et al. [40] conducted a study in the rats to develop an electrotransfer procedure with long-term effect. A non-viral vector in combination with enhanced GFP (eGFP) for evaluating the gene expression was injected in rat retinal pigment epithelium (RPE). Subjects were split into two subgroups, with or without the co-injection of the integrase plasmid from bacteriophage ΦC31. After the injection procedure, five 140-V pulses with a duration of 100 ms were applied using two electrodes (diameter of 7 mm), with the negative electrode on the injected eye and 14 mm between electrodes. The strongest gene expression was observed 48 h after delivery; however, while in the absence of integrase ΦC31 the expression nearly dropped to background levels within 3–4 weeks after treatment, the co-injection of the integrase plasmid resulted in long-term stable transgene expression throughout the 4.5-month test period.

He et al. [41] investigated an ex vivo application of gene electrotransfer to the endothelium of human corneas preserved in organ culture. Two plasmids, containing the cytomegalovirus promoter and reporter genes, respectively, were electroporated in human corneas, with eight 1-Hz 100-ms pulses of 125-mA square current. To the purposes of the study and in order to cover the entire endothelial surface, two custom-designed convex electrodes were used. Specifically, the two electrodes were placed facing each other with a complementary concave surface (8.40 mm radius of curvature); the electrodes, both with a diameter of 13.0 mm, were parallel and 2.50 mm apart, to ensure a homogeneous electric field. A screw thread on the anode shaft allowed adjustment of this distance. They reported that all electroporated corneas carried transfected corneal endothelial cells, whereas the controls carried none. Transfection efficiency determined on 63 β-gal transfected corneas ranged from 0.1 to 54% of transfected corneal endothelial cells. Electroporation produced low early corneal endothelial cells death. Anti ZO-1 staining revealed no dramatic change in corneal endothelial cells mosaic continuity, neither 1 and 3 nor 28 days after electroporation.

Touchard et al. [42] have developed a non-viral gene therapy method based on the electrotransfer of the plasmid into the ciliary muscle. These easily accessible smooth muscle cells could be turned into a biofactory for any therapeutic protein to be secreted in a prolonged fashion into ocular media.

The electrical conditions, electrode design, plasmid formulation, method and number of injections were optimized in vivo in the rat and the rabbit models. In the rat, the electrotransfer was performed with eight square wave electrical pulses of 15 V of voltage, 20 ms of duration and of 5 Hz of frequency, while in the rabbit, eight electrical pulses of 20 V square wave voltage (20 ms duration and 5 Hz frequency) were delivered. Both the models used a platinum/iridium wire electrode inserted into the preformed corneal or transcleral tunnel as cathode, and a semi-annular platinum/iridium sheet placed on the scleral surface (in front of the active cathode) as the return anode electrode. The cathode was isolated by Teflon along its entire length, except for the terminal part, which was left naked. The two models differ in the dimensional specifications of the electrodes; indeed, in the rat model a cathode with 250 μm of diameter and a non-insulated part of 1 mm was used, while in the rabbit, the cathode had a diameter of 500 μm and a non-insulated part of about 1 cm, and the anode was approximately 2 × 0.6 cm. The results obtained demonstrated that the electrotransfer of the plasmid to the ciliary muscle with a suitable medical device represents a promising system of local and prolonged protein release for the treatment of diseases of the posterior segment, avoiding repeated intraocular injections.

Kowalczuk et al. [43] performed a study to understand the role of pro-angiogenic activity of placental growth factor (PFG) in diabetic retinopathy and the effect of a sustained PGF over-expression in rat ocular media, using ciliary muscle electrotransfer of a plasmidencoding rat PGF-1 (pVAX2-rPGF-1). With the same electric protocol and electrode used on rats by Touchard et al. [42], they observed that, after fifteen days of rPGF-1 over-expression in normal eyes, tortuous and dilated capillaries were observed. At one-month, micro aneurysms and moderate vascular sprouts were detected in mid retinal periphery in vivo and on retinal flat mounts. At later stages, retinal pigmented epithelial cells demonstrated morphological abnormalities and junction ruptures. In diabetic retinas, PGF expression rose between 2 and 5 months, and one month after ET, rPGF-1 over-expression induced glial activation and proliferation.

In a further study, Touchard et al. [44] developed a novel transfection method in vivo in the adult rat. The model was called suprachoroidal electrotransfer and combined the administration of nonviral plasmid DNA into the suprachoroidal space with the application of an electrical field. In order to assess the efficacy of the method, two devices were compared. Device 1 was made of a semi-annular platinum/iridium sheet anode, placed on the scleral surface located above the inoculated area, and a semi-annular platinum/iridium wire cathode, positioned on the scleral surface, at the base of the posterior pole of the eye. Device 2 was made of a contact curved platinum/iridium sheet anode, attached to the sclera located above the injected area, and a semi-annular platinum/iridium sheet cathode, placed on the sclera, facing the anode at the opposing side of the eye. Pulses with a length of 20 ms and a frequency of 5 Hz were delivered while using both the devices; however, a reference electric field of 40 V/cm was established with the device 1, while the device 2 was used to investigate electric fields with 14, 30, 60, and 120 V/cm of intensity respectively. Choroidal cells, retinal pigment epithelial cells, and potentially photoreceptors, were efficiently transduced for at least a month when using a cytomegalovirus promoter. No ocular complications were recorded by angiographic, electroretinographic, and histological analyses, demonstrating that under selected conditions the procedure is devoid of side effects on the retina or the vasculature integrity. Moreover, a significant inhibition of laser induced-choroidal neovascularization was achieved 15 days after transfection of a soluble vascular endothelial growth factor receptor-1 (sFlt-1)-encoding plasmid. Application of electrical field intensities of 30, 60, and 120 V/cm significantly enhanced β-galactosidase expression compared to eyes treated by injection alone. Transfection efficacy increased with increasing electrical field intensities, with better efficiency obtained with 60 V/cm.

In the last included study, Touchard et al. [45] proposed a non-viral gene therapy using plasmid electrotransfer in the ciliary muscle of the rat for sustained production of secreted therapeutic proteins into the eye. The cathode (an iridium/platinum wire electrode) was inserted into the transscleral tunnel, and the anode (a semi-annular stainless-steel sheet electrode) was placed on the sclera, facing the wire electrode. A sequence of 8 pulses of 15 V (200 V/cm), 10 ms pulse duration and 180 ms (5 Hz) interval between pulses was delivered to electrotransfer the pEYS606, a clinical-grade plasmid DNA. As a result, the procedure significantly reduced ocular inflammation in two well-established rat models of uveitis, the endotoxin-induced uveitis and the experimental autoimmune uveitis (EAU). In addition, in the latter, a significant protection of photoreceptors was demonstrated after pEYS606 treatment.

## 4. IGEA Experience in the Project “Transposon-Based, Targeted Ex Vivo Gene Therapy to Treat Age-Related Macular Degeneration (TargetAMD)”

Age-related Macular Degeneration (AMD), a neurodegenerative disease of the retina, is a major cause of blindness in elderly people [46]. In the exudative form of AMD, high levels of vascular endothelial cell growth factor (VEGF) and low levels of pigment-epithelial derived factor (PEDF), an inhibitor of vascularization and a neuroprotective factor produced by retinal pigment epithelial (RPE) cells, result in subretinal neovascularization and retinal pigment cell degeneration. The current treatment by monthly injections of anti-VEGF antibodies is only effective for about the 30% of patients. To avoid the severe side effects, high costs and the overall continuing burden on health care associated with monthly antibody injections, inducing a higher level of PEDF expression to inhibit neovascularization would be a viable therapeutic alternative. Sleeping Beauty (SB) transposon technology offers a novel, safe and efficient alternative to viral approaches for gene transfer. Due to the stable chromosomal insertion of the transgene mediated by SB100X, these systems result in robust, long-term expression of the integrated transgene. SB100X shows efficient and safe transposition in human cells, and in preclinical models SB100X-mediated gene delivery has shown long-term transgene expression [47]. Transposon-based, targeted ex vivo gene therapy to treat age-related macular degeneration (TargetAMD) has shown to genetically modify iris pigmented epithelial (IPE) cells or retinal pigmented epithelial (RPE) cells that overexpress PEDF to provide a long-lasting cure of AMD. Stable PEDF gene delivery was achieved using the non-viral Sleeping Beauty transposon system, which combines the efficacy of viral delivery with the safety of naked DNA plasmids [48,49].

Even though electroporation is a standard technique in gene delivery, a modified electroporation device, cell holding cuvettes, electrodes and buffers were developed specifically for the transfection of the low cell numbers that can be isolated from the patient’s iris biopsies or pigment epithelium. Extensive in vitro and in vivo safety and efficacy studies were performed that have demonstrated that low cell numbers obtained from iris and RPE biopsies (yielding 5000 to 10,000 cells) can successfully and reproducibly transfect. Electroporation-based transfection efficiency was optimized by varying the relative amount of Sleeping Beauty transposase (SB100X) to PEDF-transposon plasmid DNA. Preliminary experiments indicate that a minimum of 5000 cells are necessary for traceable PEDF secretion after efficient transfection using electroporation. The protocol identified delivers 1 pulse with an amplitude of 200 V and a duration of 5 ms [50].

**Figure 4:**
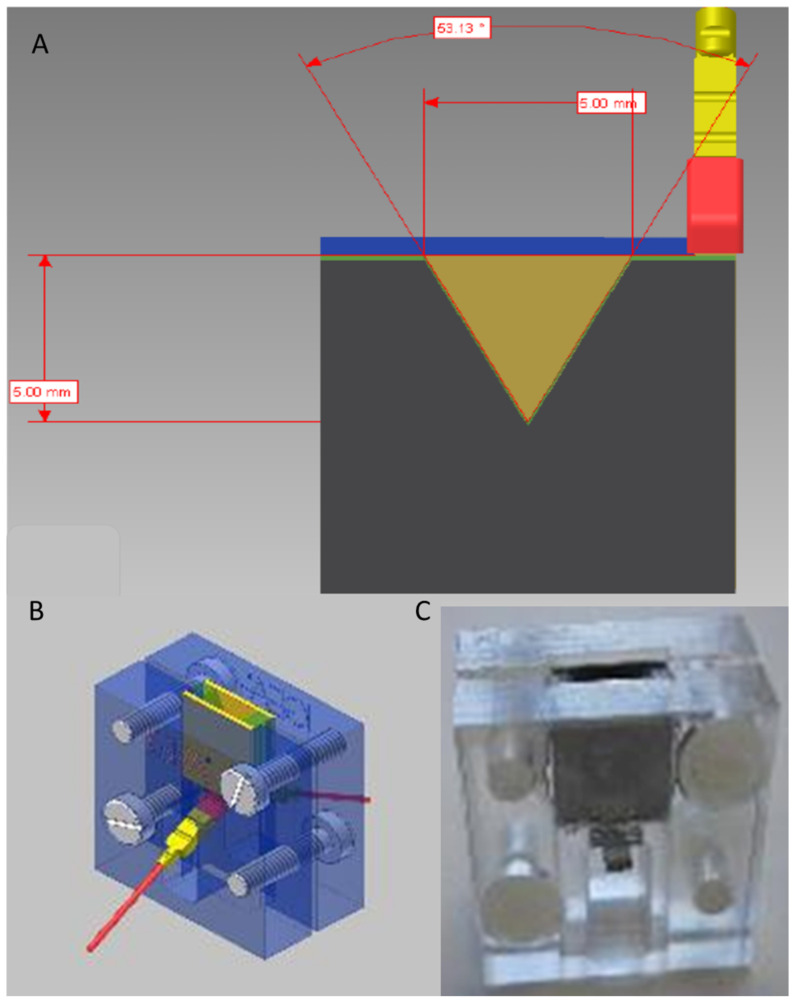
Electroporation chamber in (**A**), cuvette prototype rendering in (**B**) and real implementation in (**C**).

**Figure 5:**
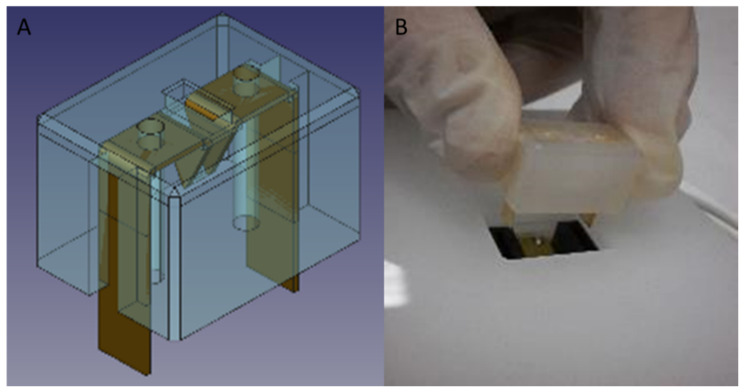
Moulded cuvette rendering (**A**) and final product (**B**).

The microcuvettes are sterile, single use, devices for GMP-grade cell product production. A V shape electroporation chamber was adopted to facilitate the cell recovery after transfection. The chamber has 5 mm aperture and 5 mm height with a distance of 2 mm between the electrodes (Figure 4), defining a maximum total volume of 25 µL. The first electrode prototypes were made of AISI 304 stainless steel while two materials, polymethylmethacrylate and polypropylene, were investigated for the body of the cuvettes (Figure 5).

This shape of the electroporation chamber ensures two different advantages: (1) a lower current density, because the area of the electrodes is bigger when compared to the overall transfection volume; (2) a lower voltage applied to the electrodes, because the electric field distribution and value depend on the distance between the electrodes; (3) to facilitate the cell recovery after transfection.

The overall results aim at optimizing the transfection process in terms of efficiency, i.e., % of transfected cells, and cell viability. To ensure the maximum biocompatibility of the cuvette, the electrodes of the final device, made of stainless steel, have been gold plated with a minimum gold thickness of 1 μm. Since the gold cannot be plated directly on the steel, a thin layer of nickel makes the interface between the two metals. Although the nickel has a low diffusion speed, creating a barrier to the diffusion of the other atoms composing the stainless steel, it might introduce biocompatibility questions. However, the limited contact time and the final product qualification according to ISO 10993-1 mitigate the risk of metals release into the tissue, ensuring biological safety. The final cuvette (Figure 5) was produced using polypropylene and injection molding technology, ensuring the biocompatibility of the device, the tolerability of the gamma-ray sterilization and the electrical safety. The electrodes have been « U » bended to have a terminal usable for the connection and the other terminal used as electroporation electrodes with a triangular shape. In this way the electrodes were completely drowned into the plastic material avoiding any leakage of liquid from the electroporation chamber.

The electroporator and the electroporation cuvettes developed for the electroporation of small volume cell samples was effective to transfect the isolated and transfected iris pigment epithelial (IPE) cells with a plasmid mixture [48].

The efficacy of the TargetAMD approach was validated under clinical trial conditions. Using GMP-grade plasmids, buffer, the microcuvette and the electroporator, rat retinal pigment epithelium (RPE) and iris pigment epithelium (IPE) cells were successfully transfected with the human PEDF gene and subretinal transplantation of the transfected cells resulted in significantly reduced Choroidal neovascularization (CNV) in a rat model of laser-induced CNV. The performance of the electroporator and electroporation cuvettes was assessed by the consortium of TargetAMD that successfully have qualified the performance of the new device with 100% reliability.

## 5. Discussion

In the early 1990s, different electrotransfer protocols were developed to overcome both the potential risks of currently available viral gene delivery systems and the low efficiency of existing non-viral systems [51,52,53,54]. In vivo electrotransfer protocols in skin applications applied short exponentially decaying pulses preceded by the injection or the topical application of DNA [55,56,57]. Similarly, direct injection of plasmid and trains of short pulses (100 ms) were used for liver applications [58,59], while DNA electroporation with long pulses (5–50 ms) was subsequently developed to treat skeletal muscles [60,61].

The suitability of skeletal muscle cells to electrotransfer procedures is mostly due to their size, shape, and morphology [62]; electroporation of skeletal muscle has been extensively used to express a number of therapeutic and physiological genes in multiple animal models, and as a bioreactor to produce and secrete a number of proteins [63]. In contrast to the homogeneity of the muscle tissue, the heterogeneous tissue composition of the eyeball lends itself to much less predictability of geometry and distribution of electric charges [11].

Pre-treatment of the targeted muscle with hyaluronidase increases the ratio of electrotransferred muscle fibres while reducing the need for high electric voltage, hence decreasing the risk of muscle damage [64].

Electroporation parameters (i.e., electrode design, field strength, and pulse duration and magnitude) need to be specifically adapted to tissue in order to obtain optimal gene transfer. Currently, in the majority of research, a pattern for in vivo electroporation averaging 610 pulses of 20–40 ms at 1 Hz with field strength on the order of 100–200 V/cm is used [65]. Shorter pulses (100 µs) of higher electric field strength (>700 V/cm) were found to be optimal for the delivery of small anti-cancer molecules [65]. It is believed that longer pulse duration leads to increased defects in the cell membrane, thus favoring DNA internalization and transfection efficacy.

The instrumentation required for electroporation consists of a pulse generator and an “applicator”, which includes the electrodes. The electrodes geometry determines the strength, homogeneity, orientation, and shape of the electrical field, as well as the current flow and the total energy transferred to the target tissue during electroporation. Different electrodes have been developed: surface electrodes, fixed electrodes for sub-cutaneous tissue, needle electrodes for deep tissues, and electroporation catheters for hollow organs.

Despite the many benefits of non-viral gene delivery, including increased safety, decreased toxicity, decreased cost, and large transgene size capability, interest in non-viral gene therapy was limited until a few years ago, in part due to transient gene expression. In fact, the efficiency of delivering remains one of the most challenge on non-viral therapy. It seems clear that, if vector shows a good efficiency, its toxicological profile increases and, on the contrary, a good toxicological profile corresponds to low efficiency. Anatomic barriers like epithelial, endothelial cells and extracellular matrix contribute to reducing the efficiency of these methods [28]. Physical methods like electroporation appear to hold promise for removing such obstacles [66].

Eyes are immune privileged organs that have a great potential for gene therapy due to their accessibility, presence of a tight ocular barrier that prevents spread to other organs and allow to easily verifying the structure and anatomy with non-invasive methods [17,18,19].

Advances in molecular biology have led to the identification and characterization of the genetic disorders responsible for many eye diseases. This has led to the development of genetic tools aimed at inducing, regulating or repairing gene expression. Most of the molecules used for these purposes are synthetic nucleic acids such as plasmids, ribozymes, siRNAs and oligonucleotides. The therapeutic efficacy of these molecules depends on the selection of suitable vectors and the efficient delivery of the system to target cells for their appropriation of expression in vivo. This approach can be used, if necessary, for both specific inhibition and induction of protein expression in target cells.

During the last decade, several authors have demonstrated the potential benefits of electrical fields (electroporation and iontophoresis) to enhance gene delivery and improve cell and tissue targeting.

Some studied showed that low-voltage electric pulses successfully transfer the gene of interest to highly selective areas of the corneal endothelium without inducing any pathological changes. These authors surmised that improved versions of this highly targeted technique would allow effective gene transfer to other intraocular tissues such as retina [32,33,34,35,36,37,38,39,40,41,42,43,44,45]. At the best of our knowledge, to date, the electrically assisted ocular gene therapy was investigated exclusively in preclinical models: in vivo and ex-vivo.

These studies have confirmed the promising potential of powerful non-viral physical methods to increase nucleic acid release in ocular tissues. These methods can potentially also provide a means for specific aiming of multiple eyepieces fabrics using custom designed plasmids or oligonucleotides.

Electrotransfer is very efficient for plasmid DNA transfection. Many attempts such as modification of electric field, buffers and electrodes have been made and the optimization of electric fields setting seems to be very important [30]. Many types of electrodes have been developed, and different materials have been used for electrodes production [67]. Using this technique, gene replacement can be designed in cases of retinal inheritance or corneal disease. It can also be used to induce the local production of therapeutic proteins such as neurotrophic growth factors, soluble receptors or specific antibodies. Therefore, a wide range of human eye diseases could, in the future, benefit from them from these gene therapy technologies.

Aim of the project TargetAMD was to introduce a novel gene therapeutic treatment that will be effective, safe and lasting for the life of the patient. TargetAMD will use subretinal transplantation of genetically modified RPE or iris pigment epithelial (IPE) cells genetically modified to overexpress the anti-angiogenic and neuroprotective factor pigment epithelium. The success of the gene therapeutic treatment proposed in TargetAMD rests on the use of the non-viral, hyperactive Sleeping Beauty (SB100X) transposon system to deliver the PEDF gene to RPE and IPE cells. The PEDF-transfected cells will then be transplanted to the subretinal space of AMD patients.

Therefore, although the basic instrumentation and its long-term application, the modalities of safety and efficacy, as well as the side effects of electrically assisted ocular gene therapy have been defined, clinical trial shall be planned and conducted in order to assess feasibility and efficacy of electrically assisted ocular gene therapy in clinical practice. Two phase Ib/IIa clinical trials (10 patients/trial) will be performed, one trial in which PEDF-transfected autologous IPE cells (UNIGE, Switzerland) and one in which autologous RPE cell transfected with the PEDF gene (KAR, Austria) will be transplanted subretinally in neovascular AMD patients. The comparison of study results could show if the use of easier to transfect, but more invasive RPE cells is superior to the use of easy to biopsy IPE cells, which presents a significantly lower risk for the patient.

## 6. Conclusions

Electrotransfer is very efficient for transfection of plasmid DNA and using this technique, gene replacement can be designed in cases of retinal inheritance or corneal disease. Therefore, a wide range of human eye diseases could, in the future, benefit from these gene therapy technologies.

## Figures and Tables

**Table 1:** Summary table of the studies analyzed in this manuscript.

	Study Design							
Manuscript	Study Information		Shape and Material of the Electrode(s)		Pulse Number and Duration	PAUSE Duration/Pulse Frequency	Applied Voltage/Current Electric Field	Results
Oshima et al. [32,33,34]	rat in vivo	corneal endothelium	ring-shaped (distance of 0.5 mm)	stainless steel	8 pulses 50 ms	Not reported	from 5 to 40 V/cm	β-Galactosidase strongest expression at 20 V. Expression limited to corneal endothelial cells. No cell damage or inflammation detected.
Sakamoto et al. [35]	rat in vivo	corneal endothelium	circular (Ø of 0.5 mm)	stainless steel	8 pulses 50 ms	80 ms	Not reported	Presence of plasminogen activator for 4 days. Significant decrease in fibrin formation. No histological damage detected.
Oshima et al. [36]	Rat in vivo	corneal stroma	bipolar linear (gap of 10 mm)	stainless steel	8 pulses 50 ms	75 ms	from 10 to 30 V/cm	Effective and selective gene transfer using eight electric pulses of 20 V for 50 ms. No cell damage or inflammation detected with voltages lower than 30 V.
Blair-Parks et al. [37]	mouse in vivo	corneal endothelium and stroma	2 golden-plated (3 mm apart)	gold	8 pulses 10 ms	Not reported	0, 100, 200, 400 V/cm	Nanogram levels of gene product expression. No trauma, edema or inflammation detected with field strength up to 200 V/cm.
Yu et al. [38]	rat in vivo	cornea	2 golden-plated (5 mm apart)	gold	8 pulses 10 ms	Not reported	200 V/cm	K5 gene effectively transfected. Effective inhibition of corneal neovascularization.
Mamiya et al. [39]	rabbit in vivo	conjunctiva	cup-shaped (not reported)	Not reported	2 × 5 pulses 50 ms	Not reported	5 V/cm	MMP-3 plasmid correctly expressed. Combination with surgery caused longer survival of the filtering bleb and decreased levels of IOP.
Chalberg et al. [40]	rat in vivo	retinal pigment epithelium	2 circular (Ø of 7 mm, 14 mm apart)	Not reported	5 pulses 100 ms	950 ms	100 V/cm	Strongest transgene expression after 48 h. 85-fold higher long-term expression achieved with co-injection of integrase.
He et al. [41]	human ex vivo	corneal endothelium	2 convex-shaped (Ø of 13 mm, 2.5 mm apart, 8.4 mm curvature radius)	stainless steel	8 pulses 100 ms	1 Hz	125 mA	eGFP expression detected in all transfected corneas.
Touchard et al. [42]	rat/rabbit in vivo	ciliary muscle	semi-annular sheet anode wire cathode (Ø of 0.25/0.5 mm)	platinum/ iridium	8 pulses 20 ms	5 Hz	15/20 V	Plasmid effectively transfected in ciliary muscle fibers. Long-lasting gene transfer (>5 months).
Kowalczuk et al. [43]	rat in vivo	ciliary muscle	semi-annular sheet anode wire cathode (Ø of 0.25 mm)	platinum/ iridium	8 pulses 20 ms	5 Hz	200 V/cm	In diabetic retinas, over-expression of rPGF-1 induced glial activation and proliferation.
Touchard et al. [44]	rat in vivo	suprachoroidal space	semi-annular sheet anode semi-annular wire cathode	platinum/ iridium	8 pulses 20 ms	5 Hz	40 V/cm	Strongest transfection efficacy at 60 V/cm (8 pulses, 20 ms, 5 Hz). No ocular complications recorded.
			curved sheet anode semi-annular sheet cathode	platinum/ iridium	8 pulses 20 ms	5 Hz	14, 30, 60, 120 V/cm	
Touchard et al. [45]	rat in vivo	suprachoroidal space	semi-annular sheet anode wire cathode	platinum/ iridium	8 pulses 10 ms	5 Hz	200 V/cm	Significant reduction of ocular inflammation observed. In EAU, significant protection of photoreceptors observed after pEYS606 treatment.

## Data Availability

All data are reported in this manuscript.
